# Screening the Community for Individuals at Clinical High Risk (CHR) for Psychosis

**DOI:** 10.1111/eip.70056

**Published:** 2025-06-03

**Authors:** Alexandre Andrade Loch, Anderson Ara, Feten Fekih‐Romdhane, Leonardo Peroni de Jesus, Julio Cesar Andrade, Melina Mendonça, Maurício Henriques Serpa, Martinus Theodorus van de Bilt, Wagner Farid Gattaz

**Affiliations:** ^1^ Laboratorio de Neurociencias (LIM 27), Instituto de Psiquiatria, Hospital das Clinicas HCFMUSP, Faculdade de Medicina Universidade de Sao Paulo Sao Paulo Brazil; ^2^ Instituto Nacional de Biomarcadores em Neuropsiquiatria (INBION) Conselho Nacional de Desenvolvimento Cientifico e Tecnológico São Paulo Brazil; ^3^ Departamento de Estatística Universidade Federal do Paraná Curitiba Brazil; ^4^ The Tunisian Center of Early Intervention in Psychosis, Department of Psychiatry “Ibn Omrane” Razi Hospital Manouba Tunisia; ^5^ Faculty of Medicine of Tunis Tunis El Manar University Tunis Tunisia; ^6^ Laboratory of Psychiatric Neuroimaging (LIM‐21), Department and Institute of Psychiatry University of Sao Paulo Medical School São Paulo Brazil

**Keywords:** perceptual abnormalities, prodromal, schizophrenia

## Abstract

**Introduction:**

The aim of our study was to assess the performance of the Prodromal Questionnaire‐16 (PQ‐16) and the Perceptual and Cognitive Aberrations scale (PCA) to screen for clinical high risk for psychosis (CHR) in a sample of nonhelp‐seeking subjects from São Paulo, Brazil.

**Methods:**

Individuals aged 18–35 years were interviewed with the PQ‐16 and the PCA. Those with a combined score > 10 on the PQ‐16 + PCA were called for assessment with the Structured Interview for Psychosis‐Risk Syndromes (SIPS). Seventy‐five individuals were deemed as CHR and 99 as healthy comparison; 44 randomly selected individuals (PQ‐16 + PCA scores < 10) joined as further controls. All participants had no DSM‐5 diagnosis. Scores of the PCA, PQ‐16 (total score and distress index), and their combinations were analysed.

**Results:**

All the proposed scorings significantly distinguished between CHR and control subjects. Considering a 7% CHR prevalence in the population, PQ‐16 score mathematically showed the best performance (AUC = 0.713), followed by the PQ‐16 score + PCA (AUC = 0.701). PQ‐16 distress had the worst performance (AUC = 0.642).

**Conclusions:**

Data provides further evidence for the use of the PQ‐16 score as an effective instrument to search for CHR states through active screening in the community. Future research should address its potential in helping CHR identification and thus reducing delays in care and minimising the risk of false positives.

## 1 Introduction

The clinical high risk concept (CHR) has been developed for almost 30 years to study the preclinical stages of schizophrenia spectrum disorders (Yung and McGorry 1996). Currently, the corpus of knowledge about such subthreshold states is substantial (Oliver et al. 2020), and preventive initiatives are spread among several countries worldwide (Loch et al. 2023; de Salazar Pablo, Estradé, et al. 2021). However, psychosis is typically associated with a long duration of untreated illness (Birnbaum et al. 2017), as psychotic phenomena entail by definition at least a certain degree of lack of self‐awareness. As such, enrollment of CHR subjects for treatment and research purposes is still a challenge and has become an important topic of discussion.

Considering the fact that transition to psychosis rates have seen a decline since the first CHR studies in the 90's (Formica et al. 2022), the field has laid eyes on the diverse recruitment strategies used to find CHR individuals (de Salazar Pablo, Estradé, et al. 2021). Services with outreach strategies and which rely on referrals may miss several at‐risk individuals, as CHR subjects avoid seeking help due to the nature of their psychotic symptoms (Allan et al. 2021; Howes et al. 2021). On the other hand, active community screening based on epidemiological surveys generates many false‐positives and entails the risk of stigma and unnecessary treatment (Mendonça et al. 2024). A systematic review observed that the prevalence of CHR in community settings is as low as 1.7% (de Salazar Pablo, Woods, et al. 2021). However, psychotic phenomena in the general population is common, with mean prevalence rates of 8% (van Os et al. 2009). This discrepancy is, for instance, one of the many causes that generates a great risk of false positives in the CHR diagnosis.

As such, screening tools have received increased attention from researchers (Kline and Schiffman 2014). One frequently used screening instrument is the Prime Screen—Revised (PS‐R) (Kline and Schiffman 2014). The PS‐R comprises 12 items to be rated on a 7‐point Likert scale and elicited a sensitivity of 0.80 and a specificity of 0.48 in a sample of 1531 Japanese participants aged 16–30 years (Kobayashi et al. 2011; Kline et al. 2012). The Youth Psychosis At‐Risk Questionnaire Brief Version (YPARQ‐B) is a 28‐item instrument which yielded a sensitivity of 0.65 and specificity of 0.90 in US adolescents and young adults (Kline et al. 2012). The PROD‐screen has 21 items and yielded a sensitivity of 0.80 and a specificity of 0.75 (Heinimaa et al. 2003). The Community Assessment of Psychic Experiences (CAPE) has 42 items and has also been widely used across different countries (Mossaheb et al. 2012). Other screening instruments include the 17‐item Early Recognition Inventory (ERIraos) (Häfner et al. 2004) and the 40‐item Eppendorf Schizophrenia Inventory (ESI) (Niessen et al. 2010), for instance.

The Prodromal Questionnaire (PQ), composed of 92 items, was one of the first instruments to be developed, in 2005, for use with adults and adolescents (Loewy et al. 2005). Later on, the same authors developed the Prodromal Questionnaire—Brief (PQ‐B), with only 21 items, but with a 5‐point Likert scale to indicate the degree of distress associated with each endorsed symptom (Loewy et al. 2011). The Prodromal Questionnaire—16 (PQ‐16) is an even shorter version of the PQ, with 16 items and also a distress scale (Ising et al. 2012). The PQ has been the most commonly used screening tool for psychosis worldwide (Kline and Schiffman 2014). In addition to being cost‐effective (Aceituno et al. 2019), the PQ confers the advantage of three times increasing the detection of young people at high risk for psychosis compared to standard referral methods (Rietdijk et al. 2012). Besides, the PQ was demonstrated to be applicable to both nonhelp‐seeking (Chen et al. 2016; Pierce et al. 2021) and help‐seeking (Karcher et al. 2020; Pelizza et al. 2019) populations. Along with these benefits, researchers have also established that the PQ achieved a sensitivity of 0.90 and a specificity of 0.49 with regard to CHR diagnosis (Loewy et al. 2005). The PQ‐B also yielded excellent performance figures, with sensitivity of 0.89 and specificity of 0.58. As for the PQ‐16, sensitivity of 0.87 and specificity of 0.87 was achieved in a Dutch study with 420 young adults seeking mental health services (Ising et al. 2012). In sum, although the existing literature tends to suggest the same optimum threshold values for the PQ to be adopted based on diagnostic accuracy findings, recent evidence has found that screening cut‐offs can differ according to the population's characteristics (e.g., general mental health samples versus nonhelp‐seeking samples) (Savill et al. 2018). This highlights the need to generate data to choose the most appropriate cutoff point for a precise and accurate CHR diagnosis that is specific to the screening approach being adopted. Selecting appropriate thresholds for a given population plays a critical role in decreasing false positives and enhancing screening efficiency (Savill et al. 2018).

Another approach in screening for psychosis that has recently been adopted by a British team is the combination of two instruments to be used in online screening. McDonald et al. (McDonald et al. 2019) used the PQ‐16 together with a 9‐item scale of perceptual and cognitive aberrations (PCA) to assess basic symptoms. The PCA was developed by the authors to specifically assess basic symptoms (e.g., thought process disturbances, cognitive disturbances). In a sample of 2296 online participants, they evaluated several cut‐offs, of both scales with separated and combined scores. In this study, best area under the curve (AUC) values were achieved with a cut‐off of six points in the PQ‐16, three points in the PCA, and 10 in the combined score of both scales (AUC of 0.72, 0.69 and 0.74, respectively). The use of the PQ‐16 + PCA combination has also been validated in children and adolescents (Spillebout et al. 2023).

As part of the Subclinical Symptoms and Prodromal Psychosis (SSAPP) project, which is a CHR cohort study established in the city of Sao Paulo, Brazil, this study aimed to assess the performance of the PQ‐16 with the PCA to screen for CHR in an enriched general population sample.

## 2 Methods

### 2.1 Sample

This study is part of the SSAPP Project, which consists of a population‐based cohort study situated in São Paulo City, Brazil, involving individuals aged 18–35 years (Loch et al. 2022). Three waves of general population screenings were carried out, each one recruiting new individuals for the study (first: 2016–2017; second: 2020; third: 2021–2022). A total of over 6500 subjects were approached. Individuals were interviewed by telephone (second wave) and face‐to‐face (first and third waves), using either the PQ (Loewy et al. 2005) (first wave) or a combination of the PQ‐16 (Ising et al. 2012) and the 9‐item scale of PCA (Schultze‐Lutter et al. 2010) (second and third waves), following previously published screening procedures (McDonald et al. 2019) (see below for details on the instruments). The current study considers data from the second and third waves, which used the PQ‐16 + PCA.

Individuals with a combined score > 10 on the sum score of the PQ‐16 + PCA scales (*n* = 174) were called for face‐to‐face interviews at the Institute of Psychiatry, University of Sao Paulo, Brazil. All individuals of the sample were assessed with the Structured Interview for Psychosis‐Risk Syndromes (SIPS) (McGlashan et al. 2001; Diniz et al. 2021) for CHR status, and with the Structured Interview for DSM‐5 diagnosis (SCID‐5) (First et al. 2016, 2017). Individuals with any DSM‐5 diagnosis were excluded from the study. Further details on the study procedures can be found elsewhere (Loch et al. 2017, 2019, 2021, 2022; Freitas et al. 2020).

After the clinical interviews, out of the 174 positively screened subjects, 75 were deemed as meeting criteria for CHR (‘CHR+’) and 99 as healthy comparison subjects (screening's false‐positives; named hereafter as ‘CHR−’). A third group was composed of 44 randomly selected individuals who scored below 10 points in the sum PQ‐16 + PCA, to participate as further control subjects (‘Control’). They also underwent SIPS and SCID‐5 assessment.

All participants signed an informed consent form, and the study was approved by the national ethics committee (CONEP# 1.709.439).

### 2.2 Instruments and Calculation of Scores

The PQ‐16 was developed by Ising et al. (Ising et al. 2012) from the 92‐item PQ (Loewy et al. 2005). It is comprised of 16 true‐or‐false items: nine items cover perceptual abnormalities, five unusual thought content and paranoia, and two negative symptoms (Savill et al. 2018). Endorsed items are further rated on a scale of distress ranging from 0 (no distress) to 3 (severe). The PQ‐16 can be scored by the total number of items endorsed (for this study named as ‘PQ‐16 score’) or by the sum of the distress scores (‘PQ‐16 distress’).

The PCA was developed to assess basic symptoms. Items for the PCA were generated from existing patient descriptions of cognitive and perceptual experiences (Uhlhaas and Mishara 2007) and from the Schizophrenia Proneness Instrument (SPI‐A) (Fux et al. 2013). It contains nine items, answered as ‘true’ or ‘false’, and total score is the sum of items endorsed (PCA).

### 2.3 Statistical Analysis

Sample was described in terms of percentage and number (categorical variables), and mean and standard deviation (continuous variables). *χ* ^2^ statistics were used to compare between‐group differences for categorical variables. Continuous variables were tested for normality with the Shapiro–Wilk test. Accordingly, group differences for normally distributed variables were tested with ANOVA, and for nonnormally distributed variables the Kruskal–Wallis test was used. To investigate the sensitivity and specificity of population screening for CHR, receiver operating characteristic analyses were conducted. They make pairwise comparisons to test if the score being assessed can correctly classify a subject as CHR or not. To test the model quality, from the applied instruments to the classification, the AUC metric was calculated. To ensure our sample reflects the population's reality, we adopted a 7% prevalence of CHR in the population in the current study's age range. This was based on previous works, including those of the group in which the distribution of the PQ and PQ‐16 in the general population were described (Loch et al. 2017, Loch et al. 2022; Schimmelmann et al. 2015). Following this prevalence, we employed a nonparametric bootstrap method correction (Efron 2000). In this sense, bootstrap samples were drawn separately in both classes and then combined, keeping the proportion of positives and negatives constant in every resample. The results are based on 1000 bootstrap samples. AUC describes how well the prediction separates subjects into those who are CHR from those who are not. An AUC value of 0.5 means that there is no difference and when the value equals 1 there is perfect separation of values of the two groups. AUC is a common performance metric over general predictions in balanced and unbalanced samples (Hanley and McNeil 1982; ACM Computing Surveys n.d.; Hu et al. 2018). Six scores were tested: PQ‐16 score, PQ‐16 distress, PCA, PCA + PQ‐16 score, PCA + PQ‐16 distress, PCA + PQ‐16 distress + PCA‐16 score. The maximum Kolmogorov–Smirnov (K‐S) statistic score was used to establish the best cut‐off score, and the performance of the best score was described (together with one point plus and one point minus the best score). SPSS statistics 25.0 for OS was used.

## 3 Results

Results for the CHR+, CHR− and Control subjects' comparisons are presented in Table 1.

**TABLE 1:** Sample characteristics.

	CHR+ (*n* = 75)	CHR−(*n* = 99)	Control (*n* = 44)	*p* *
Age	26.6 (4.21)	26.8 (4.49)	27.1 (4.09)	0.818
Gender (male)^a^	38.7% (29)	36.4% (36)	50.0% (22)	0.296
Marital status (single)^a^	81.3% (61)	83.8% (83)	72.7% (32)	0.111
Employed (yes)^a^	72.0% (54)	75.8% (75)	84.1% (37)	0.325
Years of Education (13+)^a^	49.3% (37)^b^	57.6% (57)^b^	84.1% (37)^c^	0.004
PQ‐16	10.09 (3.09)^b^	9.05 (2.99)^b^	3.14 (2.35)^c^	< 0.001
PCA	6.49 (1.76)^b^	6.39 (1.55)^b^	3.25 (1.80)^c^	< 0.001
PQ‐16 + PCA	16.59 (4.19)^b^	15.44 (3.95)^b^	6.39 (3.56)^c^	< 0.001
PQ‐16 + PCA + stress	40.56 (19.10)^b^	35.81 (16.03)^b^	12.63 (10.58)^c^	< 0.001

^a^ Described in percentage (number); where there is no indication, described in mean (standard deviation).

^b^ Means that do not share a superscript significantly differ at *p* < 0.05.

^c^ Means that do not share a superscript significantly differ at *p* < 0.05.

* ANOVA *p* value.

**FIGURE 1:**
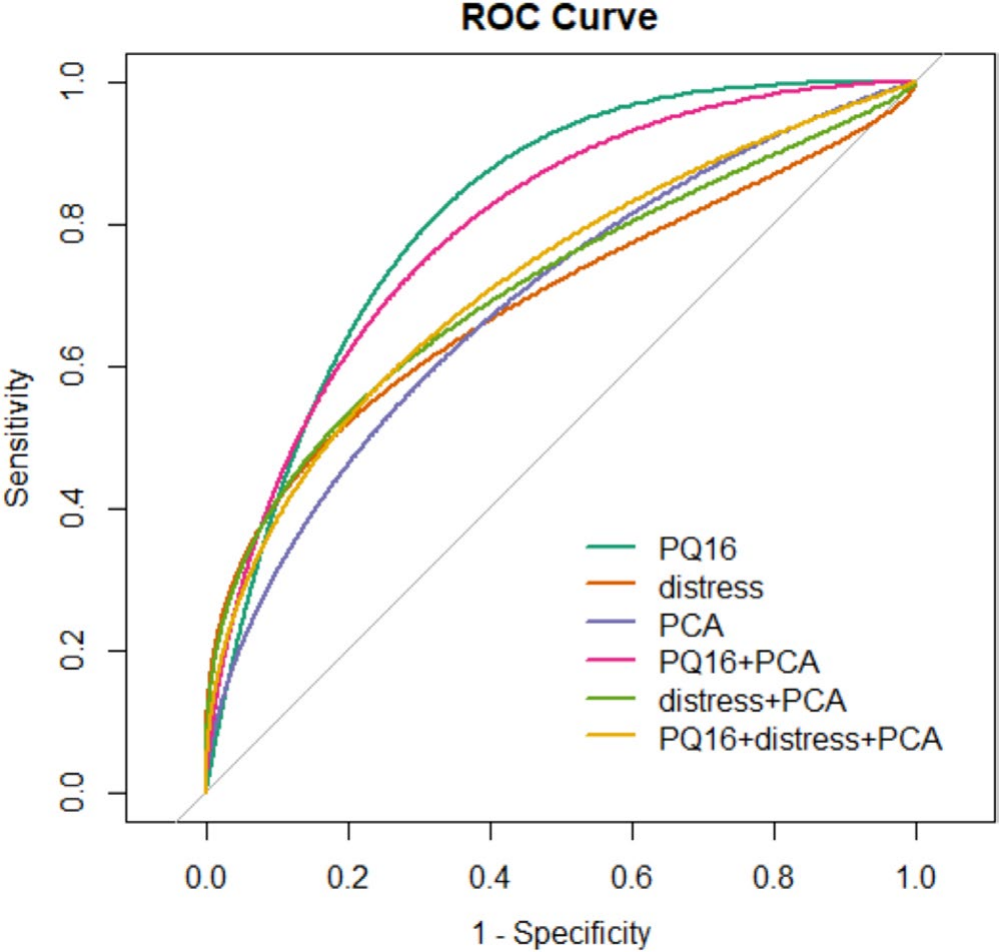
Receiver operator curve (ROC).

**FIGURE 2:**
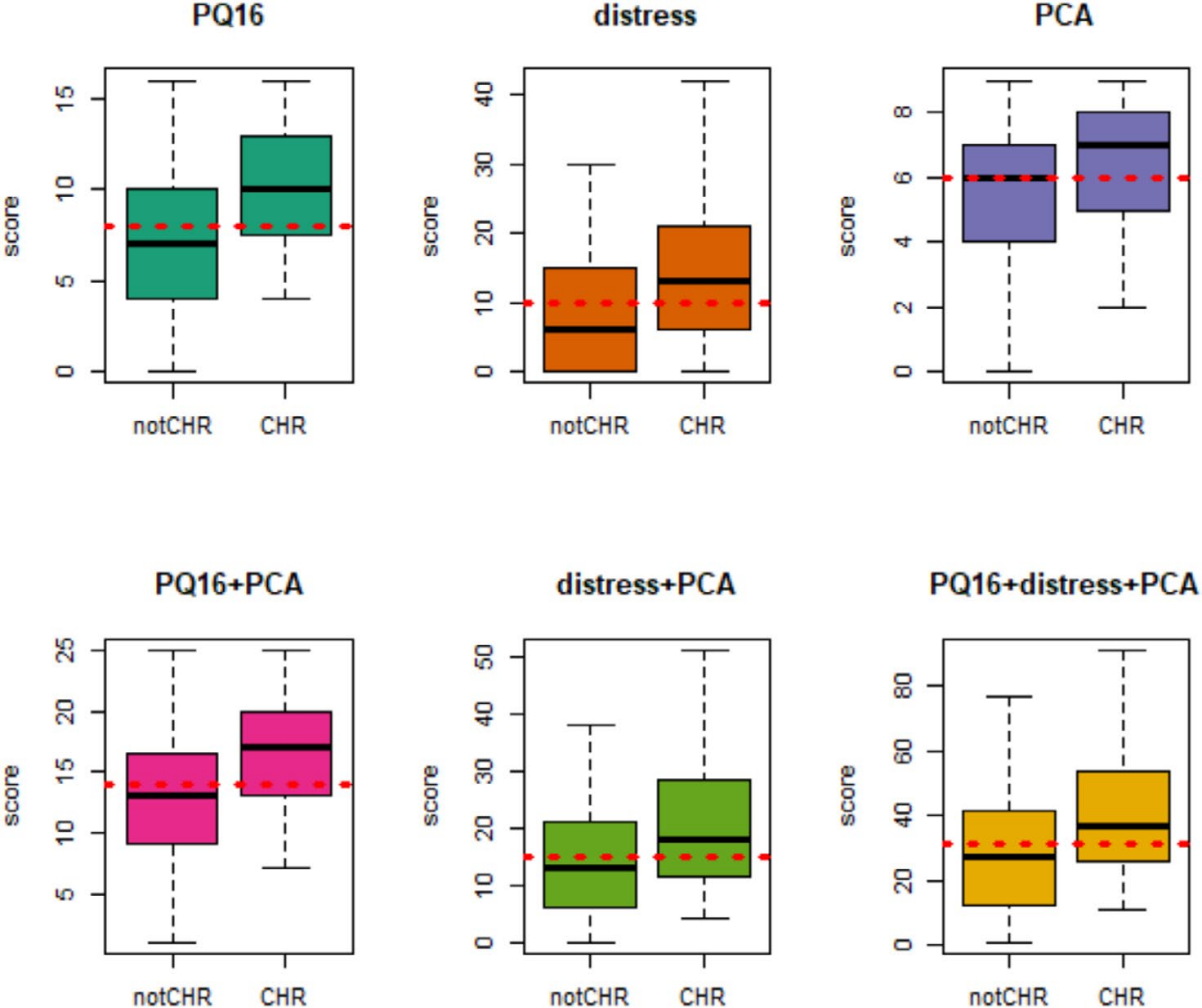
Score distributions in original sample with optimal cut‐offs (in red dashed line).

ROC metrics for PQ‐16 score, PQ‐16 distress, PCA and combinations of them are depicted in Table 2. All six proposed scorings were able to distinguish between CHR and control subjects. PQ‐16 score showed the best performance, with an AUC of 0.713, followed by the combination of PQ‐16 score + PCA, with an AUC of 0.701 (Table 2). Figure 1 displays the ROC for the six proposed scorings and Figure 2 displays the score distributions with optimal cut‐offs in the original sample.

**TABLE 2:** Area under the curve metrics (median and 95% CI).

Measure	Min–Max	Optimal cut‐off	Sensitivity	Specificity	AUC
PQ‐16 score	0–16	8 (5–13)	0.867 (0.400–1.000)	0.542 (0.285–0.906)	0.713 (0.568–0.819)
PQ‐16 distress	0–48	10 (4–26)	0.733 (0.267–0.933)	0.591 (0.384–0.970)	0.642 (0.491–0.772)
PCA	0–9	6 (4–9)	0.733 (0.084–1.000)	0.559 (0.192–1.000)	0.643 (0.404–0.757)
PQ‐16 score + PCA	0–25	14 (10–19)	0.867 (0.467–1.000)	0.542 (0.275–0.867)	0.701 (0.555–0.806)
PQ‐16 distress + PCA	0–57	15 (7–32)	0.800 (0.267–1.000)	0.559 (0.296–0.951)	0.670 (0.484–0.792)
PQ‐16 score + PQ‐16 distress+ PCA	0–73	31 (13–57)	0.800 (0.333–1.000)	0.552 (0.241–0.946)	0.675 (0.484–0.790)

## 4 Discussion

The current study examined the performance of two scales, the PQ‐16 and the PCA, in the detection of CHR from an enriched sample of nonhelp‐seeking subjects. Our data showed that the best results were achieved with the PQ‐16 score. It was better than the PQ‐16 distress, the PCA, and combinations of these scales. According to our study, the best cut‐off score would be 8, yielding a sensitivity of 0.867 and specificity of 0.542.

Contrary to a similar study conducted by McDonald et al. (McDonald et al. 2019), which used the PQ‐16 score + PCA through a self‐report website, our study examined the performance of both scales as applied by an interviewer, either face‐to‐face or by telephone. While McDonald's best results were obtained with a combination of PCA + PQ‐16 score, yielding an AUC of 0.74, our best result was achieved with the PQ‐16 score alone. AUC of our PQ‐16 score and of McDonald's PQ‐16 score were similar, 0.713 and 0.72, respectively, however with different optimal cut‐off score, 8 and 6, respectively. Consistent with our findings, a systematic review of diagnostic accuracy studies assessing the PQ indicated that the PQ is ‘an accurate predictor of CHR diagnosis’, and that ‘higher screening cut‐offs appear to be required in nonhelp‐seeking samples relative to general mental health samples’ (Savill et al. 2018).

Also importantly, our study demonstrates that the distress score did not perform well as a screener and also did not improve the performance of the PQ‐16 alone. This is in line with previous studies that showed that attenuated psychotic symptoms do not contribute significantly to help‐seeking behaviour (Kobayashi et al. 2011). Actually, psychotic symptoms entail a lack of judgement about perceptual disturbances and delusional ideas, constituting a barrier toward seeking help. As such, only trained professionals may perceive subtle reality distortions related to subclinical psychosis. This reinforces the importance of accurate instruments to be used for screening, and the debatable utility of distress scores associated with endorsed items.

At last, the PQ and abbreviated versions have been used in previous studies to detect CHR participants in secondary mental health settings (Ising et al. 2012; Savill et al. 2018; Therman et al. 2014). Our results showed that it has the potential to be used in nonhelp‐seeking individuals coming from the community, with similar performance as compared to other studies. It should be highlighted that the PQ‐16 showed the best performance, but results' 95% CI for all scales greatly overlapped. So, the PQ‐16 would not be statistically superior to the other scales, but it would be the best choice given the smaller number of items plus a tentative best performance. Overall, our findings provide support to the claims that caution should be exercised when selecting score thresholds to screen for psychosis in different populations and study designs, and further research is warranted in general population settings (Savill et al. 2018).

### 4.1 Study Limitations

As limitations of our study, we point first to the modest sample size. Although many participants were screened with the instrument, due to constraints on available research resources, only a small proportion of subjects could be interviewed with the SIPS to validate the CHR diagnosis. This generated an enriched sample, with oversampling of subjects scoring higher than 10 points in the PCA + PQ‐16. Nonetheless, our total sample was almost 60% of the McDonald study (218 against 381, respectively), producing similar results. The second limitation is the use of an interviewer to apply the instrument. This could have inhibited participants from answering more abstract questions such as those of the PCA scale. This could explain why the association of the PQ‐16 with the PCA scores did not improve the screening performance. Third, as stated above, it should be noted that this is an enriched sample, as 80% of it is composed of individuals who scored above 10 points in the PQ + PCA scales, and only 20% scored below this threshold. Thus, results presented here should not be regarded as applicable to general population settings. Ideally, a general cut‐off to the population should also consider the CHR prevalence in the population in the screening procedure. As such, further studies with more balanced samples should be carried out. Fourth, we have excluded from the clinical interviews subjects with a DSM‐5 diagnosis. Also due to resource limitations, we adopted this approach to eliminate any possible chance that psychotic phenomena could be related to ongoing disorders and focus on CHR phenomena. However, this may be questionable for some researchers, as meta‐analytic estimates of comorbidity in CHR individuals are 78% (Solmi et al. 2023). If this information is taken rigidly, one could suppose that the present study's sample is from the 22% of CHR individuals who do not have comorbid psychopathology. On the other hand, it could be argued that studies included in this meta‐analysis recruit their sample mainly through referral and risk clinics. That is, individuals are already seeking help, probably due to mood symptoms (Falkenberg et al. 2015). Also, the instrument we used, the SIPS, rules out CHR state if the psychotic phenomenon is related to any DSM diagnosis. All in all, results should be interpreted in face of this caveat. At last, it is worth highlighting that the operationalizations assessed here roughly allows 50% of false positives. In practice, this still entails the risk of stigma and fear of an unreal possibility of developing a serious mental disorder. Further actions should be taken to enhance screening's precision—to decrease false positives—and to fight the stigma related to psychosis—to reduce the burden related to eventual false positives.

## 5 Conclusion

To summarise, this study provides further evidence on the use of the PQ‐16 as a screening instrument to search for CHR states. Our findings provide valuable insights into the most appropriate tool and threshold to be adopted in nonhelp‐seeking subjects when implementing screening programmes in Brazil. This can help ensure the identification of individuals who might otherwise experience significant delays in care with a sufficient level of sensitivity, while minimising the risk of false positives.

## Data Availability

Data from the current study is available upon request to the corresponding author.
